# Longitudinal associations between complementary and alternative medicine use and pain trajectories in knee osteoarthritis: a panel analysis from the Osteoarthritis Initiative

**DOI:** 10.3389/fmed.2026.1798684

**Published:** 2026-08-13

**Authors:** Doori Kim, Yoon Jae Lee, In-Hyuk Ha

**Affiliations:** Jaseng Spine and Joint Research Institute, Jaseng Medical Foundation, Seoul, Republic of Korea

**Keywords:** acupuncture, chiropractic, complementary medicine, Osteoarthritis Initiative, osteoarthritis of knee, pain management

## Abstract

**Objective:**

This study aimed to investigate longitudinal changes in knee pain and function according to complementary and alternative medicine (CAM) use, with a particular focus on acupuncture and chiropractic therapy, among patients with knee osteoarthritis (KOA).

**Methods:**

We analyzed data from the Osteoarthritis Initiative, a large longitudinal cohort of patients with KOA. CAM use, including acupuncture and chiropractic therapy, was assessed at baseline and at 2, 4, and 8 years. Changes in knee pain and physical function were measured using the numeric rating scale (NRS) and Western Ontario and McMaster Universities Osteoarthritis Index (WOMAC). Random-effects panel regression models were used to evaluate the longitudinal associations between CAM use and outcome changes.

**Results:**

Among the 3,838 participants included in the final analysis, approximately 10% reported CAM use at each visit. CAM use was significantly associated with reductions in knee pain over time (β = −0.34, *p* = 0.007). In modality-specific analyses, chiropractic therapy was significantly associated with pain reduction (β = −0.41, *p* = 0.005), whereas acupuncture showed a non-significant association with pain improvement. CAM use was not significantly associated with changes in the WOMAC scores. The combined use of acupuncture and chiropractic showed a greater, but non-significant, reduction in pain compared with single-modality use.

**Conclusions:**

CAM use, particularly chiropractic therapy, was associated with a small but statistically significant reduction in knee pain in patients with KOA. Given the modest effect size and observational design, these findings warrant further prospective evaluation of chiropractic care as a non-pharmacological option.

## 1 Introduction

Knee osteoarthritis (KOA) is a major global health concern and one of the most common degenerative joint diseases among older adults. Its prevalence is steadily increasing due to population aging and rising obesity rates (1). KOA is associated with substantial deterioration in quality of life (1) and imposes a considerable socioeconomic burden worldwide (2). Medication remains the cornerstone of KOA management; however, concerns regarding the limited long-term effectiveness and adverse effects associated with prolonged medication use have increased interest in non-pharmacological treatment strategies (3, 4).

Complementary and alternative medicine (CAM), including acupuncture and chiropractic therapy, is widely used among patients with KOA. Clinical studies have reported the effectiveness of acupuncture in KOA (5–7). In contrast, evidence supporting chiropractic therapy remains limited, and real-world longitudinal evidence on the relationship between CAM use and changes in pain and physical function is scarce.

The Osteoarthritis Initiative (OAI) is a large-scale, longitudinal cohort study of patients with KOA (8) that includes detailed information on CAM use, providing an opportunity to examine the real-world associations between CAM use and symptom trajectories. Previous OAI-based studies have focused on the determinants of CAM use; however, none have separately evaluated specific modalities, such as acupuncture and chiropractic therapy, in relation to longitudinal changes in pain and function (9, 10).

Therefore, this study aimed to investigate longitudinal changes in knee pain and physical function according to CAM use, with a particular focus on acupuncture and chiropractic therapy, among patients with KOA using panel data analyses from the OAI cohort.

## 2 Methods

### 2.1 Data source and study population

This study used OAI raw data, which are publicly available through the National Institute of Mental Health Data Archive (https://nda.nih.gov/) upon registration and approval for data use. Participants in the OAI cohort were followed up annually for up to 9 years, and information on CAM use was collected at four time points: baseline and years 2, 4, and 8.

In the present analysis, we included participants from the OAI cohort with moderate or severe KOA symptoms or radiographic evidence of KOA. The inclusion criteria were as follows: (1) men or women aged 45–79 years (2) fulfillment of at least one of the following conditions: a numeric rating scale (NRS) pain score ≥3; Western Ontario and McMaster Universities Osteoarthritis Index (WOMAC) score ≥37.5 (total score, 0–100) or Kellgren–Lawrence (KL) grade ≥1.

Participants were excluded if the baseline values of key outcomes or covariates were missing, including sex, age, body mass index, CAM use, acupuncture use, chiropractic therapy use, WOMAC score, NRS score, comorbidity index, or history of knee surgery. In addition, participants who responded to CAM-related questionnaires at one or fewer time points were excluded.

This study was approved by the Institutional Review Board of the Jaseng Hospital of Korean Medicine (Approval No.: JASENG 2026-01-002-HE001, Date of Approval: 9 January 2026).

### 2.2 Variables

The exposure variables included use of CAM, acupuncture, and chiropractic therapy. At each visit, CAM use was defined as a response of “Yes” to a question asking whether the participant had undergone any CAM therapy in the past year. Participants who reported CAM use were asked additional questions regarding specific modalities, based on which acupuncture and chiropractic users were identified separately.

In the supplementary analyses, CAM use was further categorized into pre-defined subtypes based on previous studies (9, 10). These categories were derived from a multiple-response item and are not mutually exclusive; they were used only for descriptive and supplementary purposes and were not included in the primary analyses.

The outcome variables were knee pain and physical function, assessed using the NRS and WOMAC, respectively. The NRS measures average pain intensity over the past 30 days on a scale of 0–10. The WOMAC assesses pain, stiffness, and physical function, and is converted to a normalized total score ranging from 0 to 100.

As the NRS and WOMAC scores reflect symptoms over the preceding 30 or 7 days, changes in the NRS and WOMAC scores from the previous to the current visit were used as outcome variables.

The covariates included demographic (sex, age, race, and body mass index), socioeconomic (education level, income, current employment status, marital status, smoking status, alcohol consumption, and health insurance coverage), and clinical factors (depression score, comorbidity index, presence of rheumatoid arthritis, family history, history of knee injury or surgery, KL grade, and baseline pain and function scores).

### 2.3 Statistical analysis

Baseline characteristics according to CAM use were summarized using frequencies and percentages. In addition, the prevalence of CAM, acupuncture, and chiropractic use was calculated at each visit.

Random-effects panel regression models were used to evaluate the longitudinal associations between CAM use and changes in knee pain and physical function.

The choice between random- and fixed-effects specifications is conventionally guided by the Hausman test. However, Clark and Linzer (11) argued that this test should not be regarded as a definitive criterion, noting that zero correlation between the individual effects and the covariates is rarely observed in practice and that the Hausman test tends to reject the random-effects model when the number of observations or the within-unit variation is small. They concluded that the random-effects estimator is generally preferable when the within-unit variation of the variable of interest is small or slow-changing. Given the short panel structure in our data (a mean of 1.5 observations per participant relative to a large number of individuals), we therefore retained the random-effects model as the primary analysis despite a marginally significant Hausman test (Supplementary Table 1).

Robustness to the assumed within-person correlation structure was examined using generalized estimating equations (GEE) with an unstructured working correlation matrix and robust standard errors, and results were further confirmed using a random-intercept linear mixed model. Time-varying covariates, including body mass index, were modeled using their value at each visit.

Participants with missing sex or age at baseline were excluded; other variables were included without imputation. The random-effects panel regression accommodated an unbalanced panel, using all available person-visit observations even when the number of contributed time points varied across individuals (available-case analysis). Models were estimated by feasible generalized least squares, which is valid under a missing-at-random assumption. Neither complete-case analysis nor imputation was applied.

All the statistical analyses were performed using SAS version 9.4 (SAS Institute, Cary, NC, USA) and Stata version 15 (StataCorp, College Station, TX, USA). Statistical significance was defined as a two-sided *p*-value < 0.05.

Equation 1 :


$$\begin{array}{l} Y_{ti}=\;\beta D_{ti}\;+\;\sum \beta X_{ti}+\;\mu_{i}+\varepsilon_{ti} \end{array}$$

*Y*_*ti*_: the change in the NRS or WOMAC score for individual *i* at time *t*; *D*_*ti*_: CAM use at time *t* (1 = yes, 0 = no); *X*_*ti*_: covariates including demographic, socioeconomic, and clinical factors; μ_*i*_: individual's unique effect; and ε_*ti*_: error term.

## 3 Results

### 3.1 Study population

Of the 4,796 participants in the OAI cohort, 4,094 met the inclusion criteria. After excluding participants with missing data on key outcome variables or covariates, 4,072 participants remained. Among these, 3,838 participants who responded to the CAM-related questionnaires at least twice were included in the final analysis.

During the follow-up period, approximately 10% of participants reported CAM use at each visit. Specifically, acupuncture use was reported by approximately 1.5% of participants, whereas chiropractic use was reported by approximately 7.5% (Table 1).

**Table 1:** Status of CAM use by year.

CAM use	Baseline	2 year f/u	4 year f/u	8 year f/u
CAM use	403 (10.5)	420 (11.9)	408 (10.6)	380 (9.9)
acupuncture	60 (1.56)	67 (1.75)	63 (1.64)	61 (1.59)
chiropractic	297 (7.74)	299 (7.79)	296 (7.71)	276 (7.19)
CAM categories				
Alternative medical systems	109 (2.84)	87 (2.27)	73 (1.9)	67 (1.75)
Mind-body interventions	524 (13.65)	498 (12.98)	453 (11.8)	450 (11.72)
Manipulation and body-based methods	346 (9.02)	365 (9.51)	349 (9.09)	346 (9.02)
Energy therapies	220 (5.73)	143 (3.73)	99 (2.58)	79 (2.06)
Topical biologically based therapies	935 (24.4)	694 (18.1)	627 (16.34)	583 (15.19)
Biologically based diet or supplements	341 (8.88)	442 (11.52)	378 (9.85)	433 (11.28)

(1) alternative medical systems (e.g., homeopathy, acupuncture); (2) mind–body interventions (e.g., pilates, spiritual activities, relaxation therapy); (3) manipulation and body-based methods (e.g., massage and chiropractic care); (4) topical biologically based therapies, including rubs (e.g., tiger balm); (5) biologically based diets; and (6) biologically based supplements (e.g., glucosamine and chondroitin). CAM categories were assessed using a multiple-response item; participants could select more than one category. The category counts are therefore not mutually exclusive and may sum to more than the overall CAM-use count.

### 3.2 Baseline characteristics according to CAM use

The baseline characteristics stratified by CAM use are presented in Table 2. Female accounted for approximately 58% of the overall cohort and approximately 39% of the participants were aged 65 years or older. Overall, all the baseline characteristics were comparable between CAM users and non-users, with no clinically meaningful differences.

**Table 2:** Baseline characteristics according to CAM use.

Characteristics	Total	CAM use		
		None	Yes	Missing
Total (*N*)	3,838	3,430	403	5
Sex				
Male	1,603 (41.8)	1,469 (42.8)	134 (33.3)	-
Female	2,235 (58.2)	1,961 (57.2)	269 (66.8)	5 (100.0)
Age (years)				
45–64	2,331 (60.7)	2,151 (59.8)	278 (69.0)	2 (40.0)
≥65	1,507 (39.3)	1,378 (40.2)	125 (31.0)	3 (60.0)
Race				
White	3,019 (78.7)	2,675 (78.0)	340 (84.4)	4 (80.0)
Others	815 (21.2)	753 (22.0)	61 (15.1)	1 (20.0)
BMI				
< 25	823 (21.4)	734 (21.4)	88 (21.8)	1 (20.0)
25–30	1,512 (39.4)	1,362 (39.7)	147 (36.5)	3 (60.0)
>30	1,465 (38.2)	1,300 (37.9)	164 (40.7)	1 (20.0)
Education				
≤ High school	618 (16.1)	572 (16.7)	45 (11.2)	1 (20.0)
>High school	3,188 (83.1)	2,827 (82.4)	357 (88.6)	4 (80.0)
Income				
< 50 K	1,414 (36.8)	1,257 (36.6)	156 (38.7)	1 (20.0)
≥50 K	2,142 (55.8)	1,914 (55.8)	225 (55.8)	3 (60.0)
Marital				
Not married	1,262 (32.9)	1,123 (32.7)	136 (33.7)	3 (60.0)
Married	2,543 (66.3)	2,275 (66.3)	266 (66.0)	2 (40.0)
History of knee surgery				
No	2,875 (74.9)	2,595 (75.7)	277 (68.7)	3 (60.0)
Yes	963 (25.1)	835 (24.3)	126 (31.3)	2 (40.0)
Comorbidity score				
0	2,827 (73.7)	2,536 (73.9)	287 (71.2)	4 (80.0)
1	603 (15.7)	518 (15.1)	84 (20.8)	1 (20.0)
≥2	356 (9.3)	325 (9.5)	31 (7.7)	-
KL grade				
< 2	1,302 (33.9)	1,175 (34.3)	126 (31.3)	1 (20.0)
≥2	2,459 (64.1)	2,189 (63.8)	266 (66.0)	4 (80.0)

CAM, complementary and alternative medicine; BMI, body mass index; KL, Kellgren-Lawrence The five participants missing the baseline CAM response met our inclusion criterion of responding to the CAM questionnaire at two or more visits and were therefore retained in the analysis, although they do not appear in the baseline CAM strata. Denominators vary across variables because missing values (other than sex and age) were not imputed; percentages are calculated among non-missing responses. Married includes married or cohabiting individuals, not married includes divorced, widowed, or never married individuals.

Five participants who were included in the analysis (having responded to the CAM questionnaire at two or more visits) but did not respond at baseline are not shown in Table 2. In addition, because variables other than sex and age were not imputed when missing at baseline, the denominators vary across characteristics and do not sum to the total analytic sample of 3,838.

### 3.3 Longitudinal associations between CAM use and outcome change

In the random-effects panel analyses, CAM use was significantly associated with a reduction in knee pain as measured by the NRS (β = −0.34, 95% CI −0.59 to −0.10, *p* = 0.007). In modality-specific analyses, chiropractic use was significantly associated with a decrease in NRS scores (β = −0.41, 95% CI −0.69 to −0.13, *p* = 0.005), whereas acupuncture use showed a non-significant trend toward pain reduction (Table 3).

**Table 3:** Longitudinal associations between CAM use and changes in knee pain and function.

Exposure	NRS		WOMAC	
	Coef (95% CI)	*p*-value	Coef (95% CI)	*p*-value
CAM use	−0.34 (−0.59 to −0.10)	0.007	−0.75 (−1.88 to 0.38)	0.194
Chiropractic	−0.41 (−0.69 to −0.13)	0.005	−0.66 (−1.95 to 0.62)	0.312
Acupuncture	−0.15 (−0.75 to 0.44)	0.617	−0.91 (−3.64 to 1.81)	0.511
Chiropractic/acupuncture				
Only acupuncture	0.03 (−0.69 to 0.74)	0.94	0.10 (−3.17 to 3.37)	0.952
Only chiropractic	−0.39 (−0.68 to −0.10)	0.009	−0.49 (−1.81 to 0.84)	0.47
Both acupuncture and chiropractic	−0.67 (−1.72 to 0.39)	0.215	−3.23 (−7.96 to 1.50)	0.181

CAM, complementary and alternative medicine; NRS, numeric rating scale; WOMAC, Western Ontario and McMaster Universities Osteoarthritis Index; Coef, coefficient.

In analyses stratified by treatment combinations, chiropractic alone was associated with a significant reduction in NRS scores compared with non-use. The combined use of acupuncture and chiropractic was associated with a greater reduction in the NRS score; however, this association was not statistically significant.

For WOMAC scores, CAM use showed an overall trend toward improvement, but no statistically significant associations were observed.

In the supplementary analyses according to CAM categories, reductions in both NRS and WOMAC scores were observed across categories, although none of the associations reached statistical significance (Table 4).

**Table 4:** Longitudinal associations between CAM use and changes in knee pain and function according to CAM categories.

CAM category	NRS		WOMAC	
	Coef (95%CI)	*p*-value	Coef (95%CI)	*p*-value
Alternative medical systems	−0.32 (−0.88 to 0.24)	0.267	−1.13 (−3.70 to 1.44)	0.39
Mind-body interventions	−0.07 (−0.31 to 0.17)	0.557	0.10 (−1.00 to 1.20)	0.859
Manipulation and body-based methods	−0.29 (−0.55 to −0.04)	0.026	−0.51 (−1.70 to 0.67)	0.394
Energy therapies	−0.32 (−0.82 to 0.18)	0.204	−4.37 (−6.64 to −2.09)	< 0.001
Topical biologically based therapies	0.15 (−0.07 to 0.36)	0.177	0.81 (−0.17 to 1.78)	0.106
Biologically based diet or supplements	0.01 (−0.24 to 0.27)	0.915	−1.02 (−2.17 to 0.13)	0.081

In sensitivity analyses, the exposure–pain association for the NRS was consistent across the random-effects, linear mixed, and unstructured GEE models (chiropractic: −0.41, −0.41, and −0.41, respectively). The fixed-effects model yielded a point estimate of similar direction and magnitude (chiropractic −0.42) but did not reach statistical significance owing to a larger standard error. No significant associations were observed for WOMAC in any model. Detailed results are presented in Supplementary Tables 2–4.

## 4 Discussion

Using data from the large, longitudinal OAI cohort, this study found that CAM use, particularly chiropractic therapy, was significantly associated with improvements in knee pain over time.

Several clinical studies have reported the efficacy of manual therapy for KOA. A systematic review of 25 studies involving 2,376 participants found that manual therapy was more effective than exercise therapy or usual care in reducing KOA-related pain (12). Other studies have also reported superior pain relief with manual therapy compared with exercise therapy, electrotherapy, or usual care (13, 14). The observed association between chiropractic use and pain reduction in this study is generally consistent with these prior findings.

However, prior clinical studies were often limited by low methodological quality (15) and a focus on short-term outcomes, (12) resulting in inconsistent evidence regarding long-term effects. Moreover, evidence specifically evaluating chiropractic therapy for KOA is scarce, and to our knowledge, no clinical studies have directly assessed its effectiveness. In this context, the present study provides novel longitudinal evidence suggesting the potential therapeutic role of chiropractic therapy in KOA.

Although substantial evidence supports the effectiveness of acupuncture in KOA, (5–7) no statistically significant association was observed in the present study. This may be partly explained by the very low prevalence of acupuncture use (approximately 1–2% of visits), which likely limited the statistical power. In addition, the OAI data lacked detailed information on the treatment frequency, techniques, and duration, potentially obscuring the heterogeneous effects of acupuncture.

Several factors may explain the absence of significant association with functional outcomes. The WOMAC is a multidimensional measure encompassing pain, stiffness, and physical function and may be less responsive to short-term symptom changes than pain-specific measures. In addition, CAM use was assessed over the preceding year, whereas the WOMAC scores reflect symptoms during the past 7 days, potentially resulting in temporal misalignment between exposure and outcome.

Interestingly, the combined use of acupuncture and chiropractic therapy resulted in a greater pain reduction than either treatment alone, although this association was not statistically significant. This finding suggests that combining therapies with different mechanisms may enhance analgesic effects. Consistent with this hypothesis, a randomized controlled trial involving 242 participants reported greater pain relief when dry needling was used to manual therapy (16). In Korea, integrative treatments combining acupuncture and Chuna therapy have also been associated with improvements in pain and function (17) and reduced risks of surgery and opioid use (18). Together, these findings support the need for prospective studies evaluating combined acupuncture and manual therapy strategies.

This study had several limitations. First, its observational design precludes causal inferences. Second, CAM use was self-reported, thus introducing potential information bias. Third, detailed information on treatment intensity, frequency, and specific modalities was unavailable. Fourth, the relatively small number of CAM users, particularly acupuncture users, may have limited the statistical power for some analyses. Nevertheless, the longitudinal design and use of panel models allowed for the assessment of temporal associations beyond cross-sectional relationships. In addition, the observed associations, although statistically significant, were small in magnitude and below commonly cited thresholds for clinical importance on the NRS; accordingly, these findings should be interpreted as hypothesis-generating rather than as evidence of a clinically meaningful treatment effect. Finally, multiple exposures and outcomes were examined without formal adjustment for multiplicity, which further supports a cautious, hypothesis-generating interpretation.

In summary, this study used a large longitudinal cohort to evaluate real-world associations between CAM use and pain trajectories among patients with KOA. The consistent association between chiropractic use and pain reduction provides important evidence to inform non-pharmacological treatment strategies. These findings contribute to a more nuanced understanding of the role of CAM in KOA and may guide future clinical and interventional research.

## 5 Conclusions

In this large longitudinal cohort, CAM use, particularly chiropractic therapy, was associated with a small but statistically significant reduction in knee pain among patients with KOA. Given the modest effect size and observational design, these findings provide real-world evidence that warrants further prospective evaluation of chiropractic care as a non-pharmacological treatment option.

## Data Availability

Publicly available datasets were analyzed in this study. This data can be found here: the National Institute of Mental Health Data Archive (https://nda.nih.gov/).
