# Supplementary material for: Longitudinal associations between complementary and alternative medicine use and pain trajectories in knee osteoarthritis: a panel analysis from the Osteoarthritis Initiative
Source: Front Med (Lausanne). 2026 Aug 13;13:1798684. doi: 10.3389/fmed.2026.1798684 (PMC13519391; doi:10.3389/fmed.2026.1798684)
Supplement: Supplementary file 1 [file Table_1.docx]

Supplementary figure 1. Flow chart of participant inclusion and exclusion.

OAI, Osteoarthritis Initiative; CAM, complementary and alternative medicine.

Supplementary table 1. Hausman test comparing random- and fixed-effects specifications (p-values).

|  | **NRS** | **WOMAC** |
| --- | --- | --- |
| **CAM use** | 0.0409 | 0.0072 |
| **chiropractic** | 0.0439 | 0.0075 |
| **acupuncture** | 0.0352 | 0.0029 |
| **chiro/acupuncture** | 0.0737 | 0.0033 |

Values are p-values from the Hausman test.

NRS, numeric rating scale; WOMAC, Western Ontario and McMaster Universities Osteoarthritis Index; CAM, complementary and alternative medicine.

Supplementary table2. Longitudinal associations between CAM use and changes in knee pain and function: linear mixed model

|  | **NRS** | | **WOMAC** | |
| --- | --- | --- | --- | --- |
|  | **coef (95%CI)** | **p value** | **coef (95%CI)** | **p value** |
| **CAM use** | -0.34 (-0.59 to -0.10) | 0.006 | -0.68 (-1.79 to 0.42) | 0.226 |
| **chiropractic** | -0.41 (-0.69 to -0.13) | 0.004 | -0.66 (-1.92 to 0.60) | 0.302 |
| **acupuncture** | -0.15 (-0.75 to 0.44) | 0.613 | -0.58 (-3.24 to 2.09) | 0.671 |
| **chiro/acupuncture** |  |  |  |  |
| **only acu** | 0.03 (-0.69 to 0.74) | 0.942 | 0.57 (-2.65 to 3.79) | 0.729 |
| **only chir** | -0.39 (-0.68 to -0.10) | 0.008 | -0.49 (-1.78 to 0.81) | 0.463 |
| **both acu and chir** | -0.67 (-1.72 to 0.38) | 0.213 | -3.15 (-7.82 to 1.52) | 0.186 |

Coefficients (95% CI) were estimated using a random-intercept linear mixed model.

NRS, numeric rating scale; WOMAC, Western Ontario and McMaster Universities Osteoarthritis Index; CI, confidence interval; CAM, complementary and alternative medicine.

Supplementary table3. Longitudinal associations between CAM use and changes in knee pain and function: generalized estimating equations with an unstructured working correlation.

|  | **NRS** | | **WOMAC** | |
| --- | --- | --- | --- | --- |
|  | **coef (95%CI)** | **p value** | **coef (95%CI)** | **p value** |
| **CAM use** | -0.35 (-0.60 to -0.09) | 0.007 | -0.66 (-1.84 to 0.51) | 0.27 |
| **chiropractic** | -0.41 (-0.69 to -0.12) | 0.005 | -0.64 (-1.96 to 0.68) | 0.342 |
| **acupuncture** | -0.15 (-0.75 to 0.45) | 0.619 | -0.58 (-3.36 to 2.19) | 0.68 |
| **chiro/acupuncture** |  |  |  |  |
| **only acu** | 0.03 (-0.74 to 0.79) | 0.943 | 0.56 (-2.67 to 3.79) | 0.733 |
| **only chir** | -0.39 (-0.69 to -0.09) | 0.01 | -0.46 (-1.81 to 0.89) | 0.506 |
| **both acu and chir** | -0.67 (-1.42 to 0.08) | 0.08 | -3.14 (-8.39 to 2.11) | 0.241 |

Coefficients (95% CI) were estimated using generalized estimating equations with an unstructured working correlation matrix and robust standard errors. Abbreviations as in Supplementary Table S2.

Supplementary table4. Longitudinal associations between CAM use and changes in knee pain and function: fixed-effects model.

|  | **NRS** | | **WOMAC** | |
| --- | --- | --- | --- | --- |
|  | **coef (95%CI)** | **p value** | **coef (95%CI)** | **p value** |
| **CAM use** | -0.48 (-1.01 to 0.05) | 0.078 | -1.27 (-3.50 to 0.97) | 0.267 |
| **chiropractic** | -0.42 (-1.05 to 0.22) | 0.198 | -0.59 (-3.24 to 2.07) | 0.664 |
| **acupuncture** | -0.11 (-1.38 to 1.17) | 0.871 | -5.01 (-10.39 to 0.38) | 0.068 |
| **chiro/acupuncture** |  |  |  |  |
| **only acu** | -0.02 (-1.41 to 1.37) | 0.978 | -5.41 (-11.30 to 0.48) | 0.072 |
| **only chir** | -0.41 (-1.05 to 0.24) | 0.218 | -0.57 (-3.26 to 2.12) | 0.677 |
| **both acu and chir** | -0.65 (-2.84 to 1.55) | 0.563 | -4.15 (-13.30 to 4.99) | 0.373 |

Coefficients (95% CI) were estimated using a fixed-effects panel model. Time-invariant covariates are omitted by design. Abbreviations as in Supplementary Table S2.
